# Comparison emergence of sedation, using dexmedetomidine and remimazolam, in spinal anaesthesia - double blinded randomized controlled trial

**DOI:** 10.7150/ijms.95736

**Published:** 2024-06-03

**Authors:** Seung-Wan Hong, Jun-Young Park, Ka-Young Rhee, Seong-Hyop Kim

**Affiliations:** 1 Department of Anesthesiology and Pain medicine, Konkuk University Medical Center, Konkuk University School of Medicine, Seoul, Korea.; 2 Department of Infection and Immunology, Konkuk University School of Medicine, Seoul, Korea.; 3 Department of Medicine, Institute of Biomedical Science and Technology, Konkuk University School of Medicine, Seoul, Korea.; 4 Department of Medical Education, Konkuk University School of Medicine, Seoul, Korea.

**Keywords:** Sedation, Dexmedetomidine, Remimazolam, Spinal anesthesia, Hemodynamics, Respiratory rate

## Abstract

**Background:** Continuous intravenous infusion of remimazolam may be suitable for sedation in patients undergoing regional anaesthesia. However, there have been no studies comparing remimazolam and dexmedetomidine for this purpose. This study compared emergence from sedation between dexmedetomidine and remimazolam following continuous intravenous infusion in patients undergoing spinal anaesthesia.

**Methods:** This double-blinded, randomised controlled trial assessed the sedative effects of dexmedetomidine and remimazolam. Following spinal anaesthesia, patients were sedated using continuous intravenous infusion of either dexmedetomidine (D group) or remimazolam (R group).The D group received dexmedetomidine administered at 6 mL/kg/h (6 µg/kg/h) for 10 minutes, followed by 1 mL/kg/h (1 µg/kg/h). The R group received remimazolam administered at 6 mL/kg/h (6 mg/kg/h) for 10 minutes, followed by 1 mL/kg/h (1 mg/kg/h). Sedation levels were evaluated using the Modified Observer's Assessment of Alertness/Sedation (MOAA/S) scale. The time to reach MOAA/S ≤ 3 from the start of drug infusion and the time to reach MOAA/S = 5 from the end of infusion were recorded. Hemodynamic parameters and respiratory rate were also monitored.

**Results:** The R group reached MOAA/S ≤ 3 significantly faster than the D group during induction of sedation (4 ± 1 minutes and 11 ± 3 minutes, respectively, *p* < 0.001). The R group also reached MOAA/S = 5 significantly faster than the D group during emergence from sedation (11 ± 3 minutes and 16 ± 5 minutes, respectively, *p* < 0.001). Both groups maintained stable hemodynamic parameters and respiratory rate without any significant differences, although the mean heart rate was significantly lower in the D group than in the R group after the start of infusion.

**Conclusion:** Remimazolam demonstrated significantly faster induction of and emergence from sedation compared to dexmedetomidine, with no significant differences in haemodynamics or respiratory depression.

## Introduction

Although clinical outcomes have been similar, the demand for regional anaesthesia in preference to general anaesthesia has recently grown 1, 2. The need for sedation during regional anaesthesia has also increased due to patient anxiety, discomfort, and surgeons' requests.

Any intravenous anaesthetic agent can be used for sedation during regional anaesthesia, but benzodiazepines and dexmedetomidine are preferred over propofol due to a lower risk of respiratory depression 3, 4. The characteristics of sedation with midazolam, a benzodiazepine, differ from those of dexmedetomidine 5. Midazolam induces a significantly faster onset of sedation compared to dexmedetomidine 6, but it also causes more profound variations in sedation depth 5, 6. Conversely, dexmedetomidine is associated with more profound sedation and delayed recovery compared to midazolam 7.

Recently, remimazolam, a new ultra-short-acting intravenous anaesthetic, has been developed and approved. It is an ester-based benzodiazepine that is rapidly hydrolysed by tissue esterases into an inactive metabolite in a dose-independent manner. Remimazolam combines the hypnotic characteristics of midazolam with rapid emergence and minimal cardiovascular and respiratory depression 8, 9. Therefore, continuous intravenous infusion of remimazolam might be suitable for sedation in patients undergoing regional anaesthesia. However, there have been no studies comparing remimazolam and dexmedetomidine in continuous intravenous infusion for sedation in patients undergoing regional anaesthesia.

We hypothesised that continuous intravenous infusion of remimazolam for sedation during regional anaesthesia might result in earlier emergence and different sedation characteristics compared to continuous intravenous infusion of dexmedetomidine. This study compared emergence from sedation between dexmedetomidine and remimazolam following continuous intravenous infusion in patients undergoing spinal anaesthesia.

## Materials and Methods

### Study population

This study was conducted according to the guidelines of the Declaration of Helsinki. After obtaining approval from the Institutional Review Board (The Institutional Review Board of Konkuk University Medical Center, Seoul, Korea; Reference No, KUH2022-04-004; Date of Approval, June 30, 2022) and informed consents from patients, the study was registered at the Clinical Research Information Service, Korea Centers for Disease Control and Prevention, Ministry of Health and Welfare (KCT0008127; date of registration, January 17, 2023; http://cris.nih.go.kr). Patients with no adverse past medical history undergoing orthopaedic surgery under spinal anaesthesia were enrolled. Exclusion criteria were: contraindications to spinal anaesthesia, contraindications to the use of dexmedetomidine or remimazolam, urgent or emergent cases, a history of drug abuse, QT prolongation on preoperative electrocardiography, and concurrent surgeries.

Before induction of anaesthesia, patients were randomly allocated to either the D group or the R group based on opening a sequentially numbered sealed envelope containing group allocation. The allocation sequence was generated through random permuted block randomisation. All medical staff involved in patient care were blinded to the study. Data were collected by trained observers who did not participate in patient care and who were blinded to the study.

### Spinal anesthesia

A standardised anaesthetic technique was used. Patients arrived in the operating room without premedication. After routine non-invasive monitoring (pulse oximetry, electrocardiography, and systemic blood pressure monitoring), anaesthesia was induced. The anaesthesiologists, who were blinded to the study, followed the protocol described below.

Spinal anaesthesia was administered with the patient in the lateral position, with the leg for surgery placed in the dependent position. Povidone-iodine was used as an antiseptic solution for skin preparation. Hyperbaric bupivacaine 0.5% was administered intrathecally using a 25-gauge Quincke spinal needle at L4/L5 or L3/L4, following confirmation of cerebrospinal fluid. The total dose of 0.5% hyperbaric bupivacaine was determined by the attending anaesthesiologist based on the patient's condition and the type of surgery. The patient was repositioned from lateral to supine after administration of 0.5% hyperbaric bupivacaine. Sensory blockade was assessed using a pinprick test, and motor blockade was assessed using the Bromage scale. During anaesthesia, a simple mask providing a 5.0-L/min oxygen flow and continuous end-tidal carbon dioxide (ETCO_2_) monitoring via capnography were used for safety. Respiratory depression was defined as an oxygen saturation < 90%, as measured by pulse oximetry, or a respiratory rate (RR) < 10 breaths/min, as measured by capnography 10. If respiratory depression occurred, the patient's head was repositioned. If it persisted, a nasal airway was applied.

Hemodynamic stability was maintained as follows. Phenylephrine 30 µg was administered if the mean systemic blood pressure (MBP) was below 60 mmHg and the heart rate (HR) was above 40 beats/min. Ephedrine 4 mg was given if MBP was below 60 mmHg and HR was below 40 beats/min. Atropine 0.5 mg was administered if HR was below 40 beats/min. Continuous phenylephrine infusion was used if MBP remained below 60 mmHg despite repeated phenylephrine injections. After surgery, the patients were transferred to the post-anaesthetic care unit (PACU).

### Dexmedetomidine and remimazolam for sedation

A 50-mL syringe containing either dexmedetomidine 1.0 µg/mL [Precedex™ (4.0 µg/mL), New York, NY, USA] for the D group or remimazolam 1 mg/mL [Byfavo™ (50 mg/vial), Hana Pharm Co., Ltd., Korea] for the R group was prepared by a registered nurse who was not involved in patient care and was blinded to the study. The drugs were mixed in normal saline to create a total solution volume of 50 mL based on the group allocation. After confirming the spinal anaesthesia block level, administration of dexmedetomidine to the D group and remimazolam to the R group was initiated using identical syringes. Dexmedetomidine was administered to the D group intravenously at a rate of 6 mL/kg/h (6 µg/kg/h) for the first 10 minutes, followed by a maintenance rate of 1 mL/kg/h (1 µg/kg/h). Remimazolam was administered to the R group intravenously at a rate of 6 mL/kg/h (6 mg/kg/h) for the first 10 minutes, followed by a maintenance rate of 1 mL/kg/h (1 mg/kg/h). Both dexmedetomidine and remimazolam were maintained until the end of surgery.

### Measurements

The Modified Observer's Assessment of Alertness/Sedation (MOAA/S) scale was used to evaluate sedation levels 11. In both groups, the MOAA/S scale was checked every minute from the start of infusion until the MOAA/S score reached ≤ 3. The time taken was recorded. During infusion, MOAA/S scores, MBP, HR, and RR were recorded every 15 minutes for both groups, together with the total amounts of dexmedetomidine or remimazolam infused.

Anaesthesia time, from arrival in the operating room to discharge from the operating room, and surgery time, from surgical incision to skin dressing, were recorded. MOAA/S scores, MBP, HR, and RR were measured every 5 minutes from arrival in the PACU (PACU_0_) until discharge from the PACU (PACU_End_). The time to reach an MOAA/S score of 5 (T_MOAA/S = 5_) after stopping infusion was also recorded.

Postoperative pain was assessed using a visual analogue scale, with scores ranging from 0 (no pain) to 100 (the worst pain imaginable), while postoperative nausea and vomiting (PONV) were assessed using a four-point ordinal scale (0 = none, 1 = nausea, 2 = retching, 3 = vomiting) during the PACU stay. Postoperative pain and PONV were managed according to institutional protocol. Ketorolac (0.5 mg/kg) and fentanyl (0.2 µg/kg) were administered intravenously as the first and second rescue treatments for postoperative pain, respectively. Ondansetron (4 mg) and dexamethasone (5 mg) were administered intravenously as the first and second rescue treatments for PONV, respectively.

### Statistics

The primary outcome was T_MOAA/S = 5_. Based on a pilot study with 10 patients in each group, the _TMOAA/S = 5_ was 13.5 ± 3.9 minutes for the D group and 11.0 ± 2.3 minutes for the R group. Using G*Power software version 3.1.9.7 (Universität Kiel, Kiel, Germany), the sample size was calculated based on an effect size of 0.790. A sample size of 70 was determined for T_MOAA/S = 5_, with a power of 0.9 and an alpha level of 0.05 12, 13.

Statistical analyses were conducted using SPSS for Windows (version 27.0; IBM Corp., Armonk, NY, USA). Categorical variables were analysed using the chi-square or Fisher's exact test, and continuous variables were analysed using the independent *t*-test. T_MOAA/S ≤ 3_ and T_MOAA/S = 5_ were illustrated using a Kaplan-Meier curve. Data were expressed as the number of patients, means ± standard deviation, or medians (first-third quartile) according to the results of the Shapiro-Wilk normality test. For all analyses, *P* < 0.05 was considered statistically significant.

## Results

**Figure 1:**
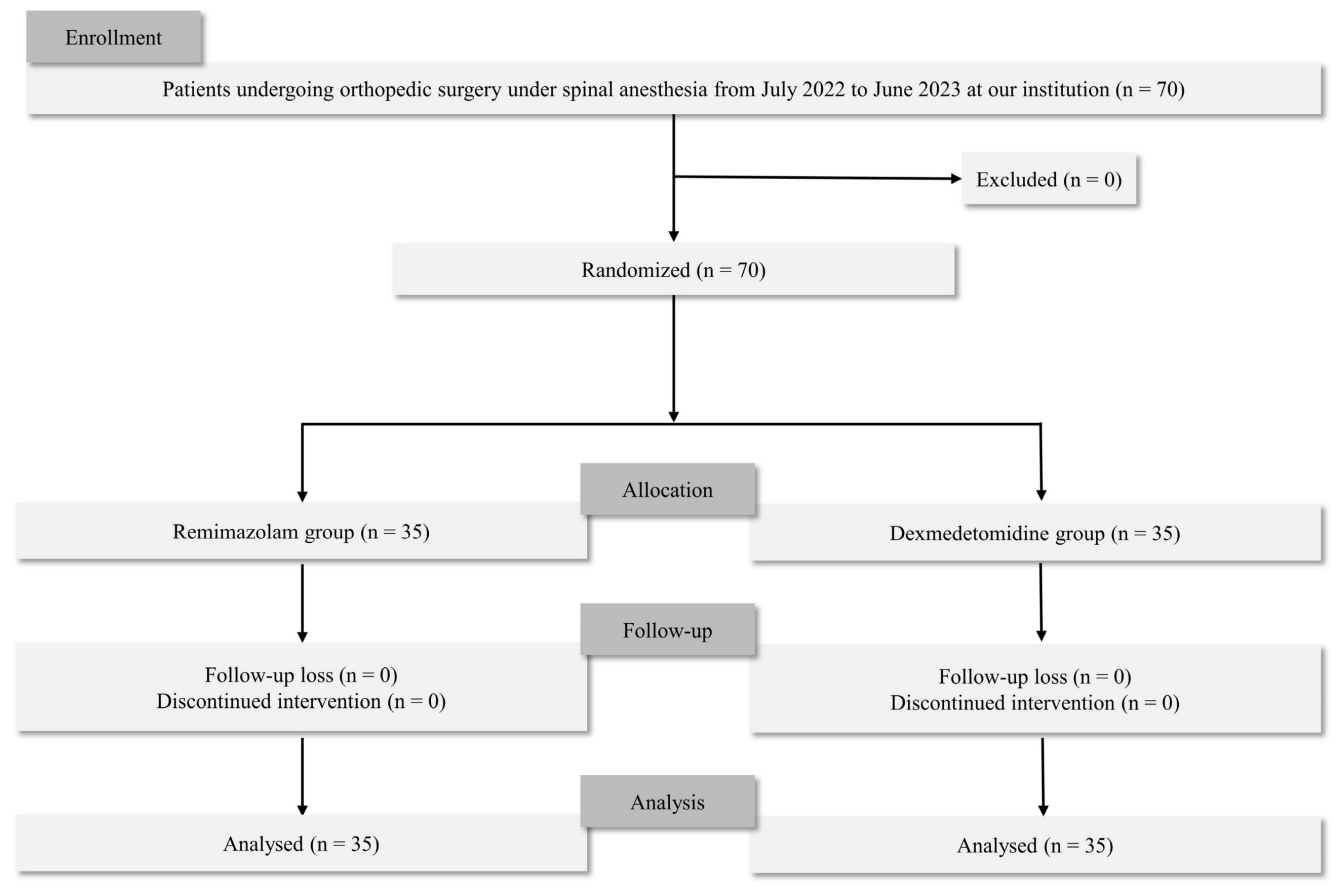
Flow of the participants for the study.

In total, 70 patients were eligible for the study, and no patient was excluded. Thus, 70 patients were included in the final analysis (Figure 1). Demographic variables were similar in the two groups (Table 1). None of the patients experienced respiratory depression during sedation in either group.

**Figure 2:**
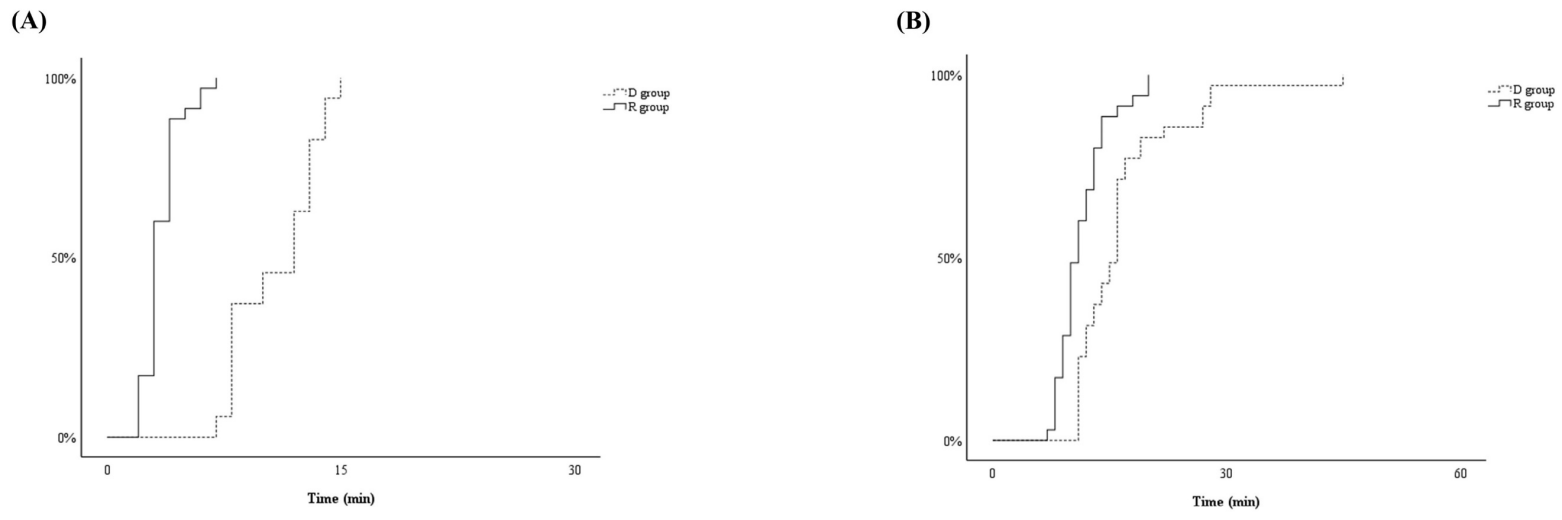
The Kaplan-Meier curves illustrating time from start for the infusion of dexmedetomidine for D group or remimazolam for R group to reach Modified Observer's Assessment of Alertness/Sedation (MOAA/S) Scale ≤ 3 (T_MOAA/S ≤ 3_) (A) and the time from stop for the infusion of dexmedetomidine for D group or remimazolam for R group to reach MOAA/S = 5 (T_MOAA/S = 5_) (B).

**Figure 3:**
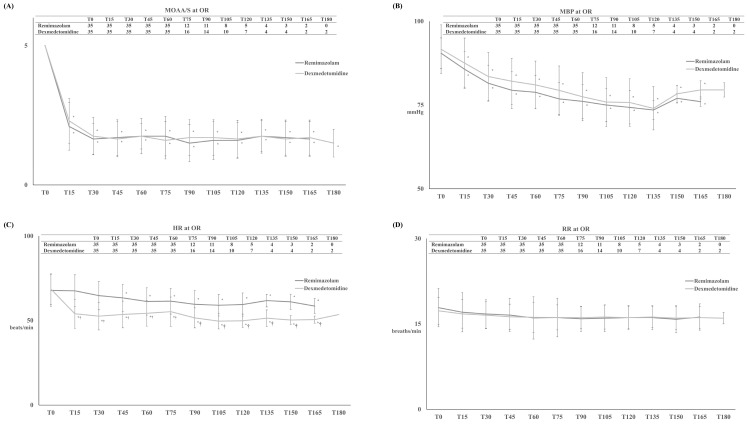
Modified Observer's Assessment of Alertness/Sedation (MOAA/S) Scale (A) with mean systemic blood pressure (MBP) (B), heart rate (HR) (C) and respiratory rate (RR) (D) during the infusion of dexmedetomidine for D group or remimazolam for R group. **Abbreviation:** T_x_, the time for the infusion of dexmedetomidine for D group or remimazolam for R group (x represents “minutes”.). Tables showed the number of patients at each time. ^*^: *p* < 0.05 compared with T0. ^†^: *p* < 0.05 compared with R group.

T_MOAA/S ≤ 3_ differed significantly between the two groups, with significantly shorter induction in the R group than in the D group (4 ± 1 minutes and 11 ± 3 minutes, respectively, *p* < 0.001) (Figure 2-A). After the start of drug infusion, MBP, HR, and RR decreased in both groups. However, MBP did not differ significantly between the two groups, remaining above 60 mmHg during anaesthesia in both groups (Figure 3-B). HR was significantly lower in the D group compared to the R group after the start of infusion (*p* < 0.001) (Figure 3-C). There was no significant difference in RR between the two groups (Figure 3-D).

**Figure 4:**
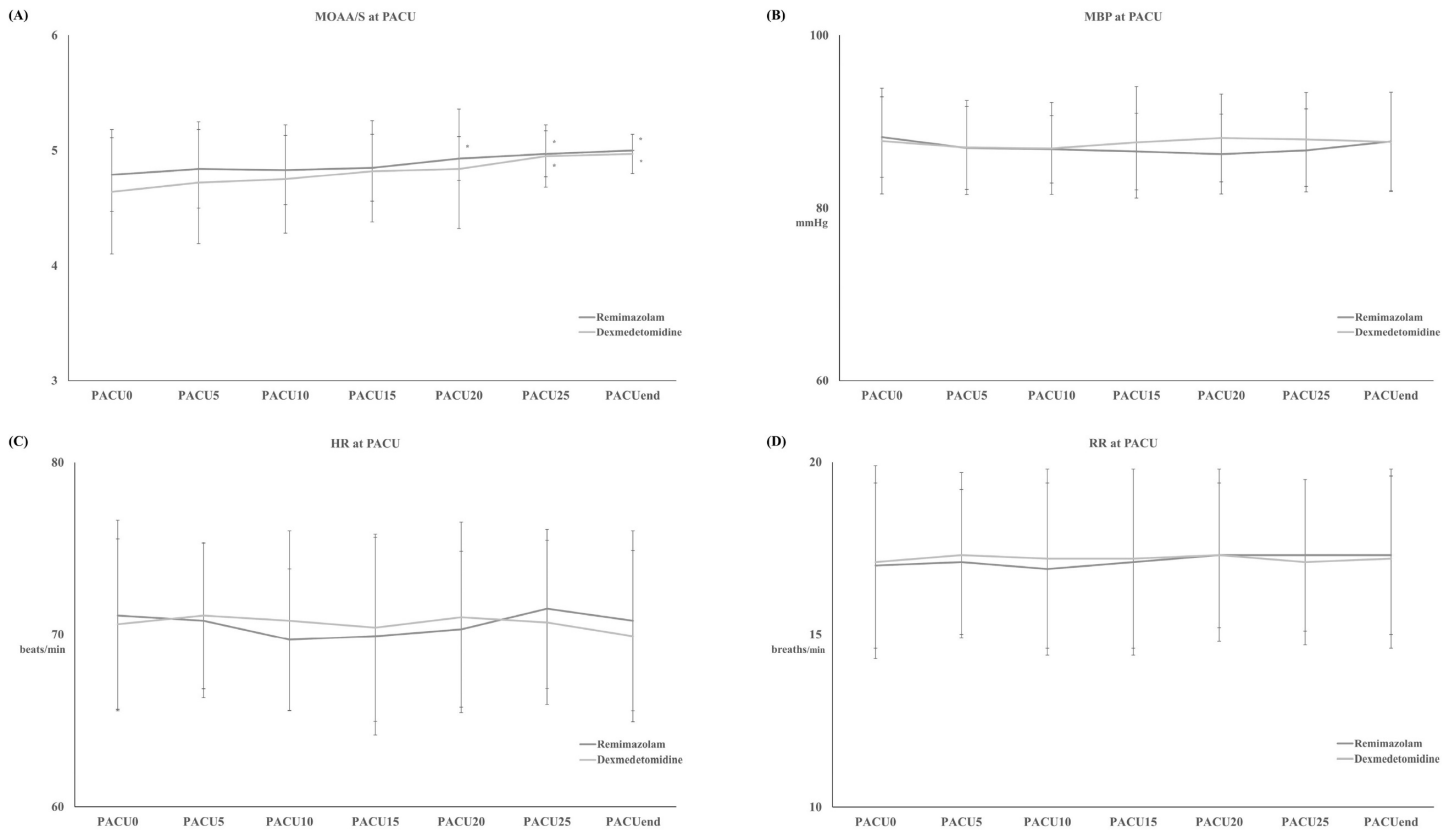
Modified Observer's Assessment of Alertness/Sedation (MOAA/S) Scale (A) with mean systemic blood pressure (MBP) (B), heart rate (HR) (C) and respiratory rate (RR) (D) during post-anesthetic care unit (PACU) stay. **Abbreviation:** PACU_0_, arrival at PACU; PACU_End_, discharge from PACU; PACU_x_, the time of stay at PACU (x represents “minutes”.). ^*^: *p* < 0.05 compared with PACU_0_.

T_MOAA/S = 5_ also differed significantly between the two groups, with the R group emerging from sedation significantly faster than the D group during (11 ± 3 minutes vs. 16 ± 5 minutes, respectively, *p* < 0.001) (Figure 2-B). There were no differences between the two groups in haemodynamics, RR, postoperative pain, or PONV during the PACU stay (Figure 4 and Table 2). None of the patients required postoperative pain management or PONV during the PACU stay.

## Discussion

This study demonstrated that both induction of, and emergence from, sedation were significantly faster with remimazolam than with dexmedetomidine during continuous intravenous infusion. Additionally, sedation with either resulted in decreased MBP without significant hemodynamic instability, although HR was significantly lower with dexmedetomidine compared to remimazolam.

While the criteria for satisfactory sedation have not been definitively decided in textbooks, several conditions are typically included: rapid induction of sedation without causing patient anxiety; sufficient depth of sedation, with hemodynamic and respiratory stability; limited movement during sedation; rapid emergence from sedation; and minimal sedative-related adverse events after emergence 14.

Dexmedetomidine is often used for sedation with continuous intravenous infusion instead of propofol, a commonly used intravenous anaesthetic, due to its remarkable hemodynamic and respiratory stability 15, 16. However, dexmedetomidine is associated with delayed induction and emergence 17, 18. In this study, remimazolam demonstrated significantly faster induction and rapid emergence compared to dexmedetomidine. Furthermore, remimazolam exhibited similar hemodynamic and respiratory changes to those of dexmedetomidine during sedation, indicating superior characteristics for sedation purposes. Recent studies have consistently demonstrated the hemodynamic and respiratory stability of remimazolam 19-21, but the studies were not specifically designed to compare remimazolam and dexmedetomidine during continuous intravenous infusion.

The present study was designed as a double-blinded randomised controlled trial to obtain robust results. However, the study design included a consideration regarding dosing strategies for continuous intravenous infusion sedation. In clinical practice, the recommended dosing for sedation involves using dexmedetomidine with a loading dose of 1 µg/kg over 10 minutes, followed by an infusion rate of 0.2-1 µg/kg/h. For remimazolam, standard practice typically involves an initial bolus of 5 mg, followed by boluses of 2.5 mg every 15 minutes, regardless of body weight 22. In this study, dexmedetomidine was administered to the D group at 6 mL/kg/h (6 µg/kg/h) for the first 10 minutes, aligning with clinical practice recommendations for a loading dose. However, remimazolam was administered at 6 mL/kg/h (6 mg/kg/h) for the first 10 minutes, which is a higher loading dose compared to standard clinical recommendations.

The significantly faster induction of sedation observed in the R group may have been attributed to this higher dosing strategy. However, it is noteworthy that none of the patients in the R group experienced hemodynamic instability or respiratory depression. Furthermore, despite the higher loading dose, the R group demonstrated significantly earlier emergence from sedation compared to the D group. The absence of respiratory depression in both groups was significant. Previous studies have indicated that prolonged administration of dexmedetomidine or remimazolam can lead to respiratory depression, although comparisons with other sedatives have been limited 23-25. Our results may be related to the relatively young and healthy patient population in this study and the frequent evaluation of the MOAA/S scale that was conducted every minute during the loading phase of dexmedetomidine or remimazolam administration.

This study had several limitations. First, the sedative effects of dexmedetomidine and remimazolam were not compared with a propofol control group. Previous studies included propofol for comparison, and doing so in this study could have provided more robust results regarding differences in the characteristics of dexmedetomidine and remimazolam. However, conducting a double-blinded controlled study would have been challenging due to propofol's distinctive colour. Second, the evaluation in this study was limited to the intraoperative period and the PACU stay. Dexmedetomidine has an analgesic effect that could have led to different postoperative pain experiences in the D group when the effects of spinal anaesthesia wore off. However, the spinal anaesthesia could have interfered with precise evaluation of postoperative pain in the two groups. Therefore, we focused on the sedative effects during only the intraoperative and PACU periods.

In conclusion, remimazolam demonstrated significantly faster induction of, and emergence from, sedation compared to dexmedetomidine, with no significant differences between the two groups in haemodynamics or respiratory depression.

## Figures and Tables

**Table 1:** Demographic data.

		D group (n = 35)	R group (n = 35)	*P* value
Gender (Female/Male)		15 / 20	13 / 22	0.808
Age (years)		38 ± 12	39 ± 15	0.508
Height (cm)		168 ± 9	170 ± 8	0.496
Weight (kg)		70 ± 10	71 ± 13	0.331
Hx of motion sickness		6	5	0.746
Hx of PONV		5	6	0.764
Spinal anesthesia level				
	Sensory block (T-spine)	8 ± 2	9 ± 2	0.725
Anesthesia time (min)		134 ± 55	130 ± 46	0.366
Surgery time (min)		101 ± 54	98 ± 40	0.827
Type of surgery				0.677
	Osteotomy	6	5	0.743
	Meniscectomy	4	5	0.721
	Meniscus repair	6	7	0.759
	Ligament repair	16	13	0.467
	Implant removal	3	5	0.452
Sedative agents				
	Dexmedetomidine (µg)	177 ± 57		
	Remimazolam (mg)		175 ± 63	
T_MOAA/S ≤ 3_ (min)		11 ± 3	4 ± 1	< 0.001
T_MOAA/S = 5_ (min)		16 ± 5	11 ± 3	< 0.001
Respiratory depression		0/35	0/35	1.000
Vasoactive agents				
	Phenylephrine^*^	2/35	3/35	1.000
	Ephedrine^†^	1/35	2/35	1.000
	Atropine (mg)	-	-	-

Data is expressed as number of patients or mean ± standard deviation. **Abbreviations:** Hx, history; PONV, postoperative nausea and vomiting; T_MOAA/S ≤ 3_, the time from start for the infusion of dexmedetomidine for D group or remimazolam for R group to reach Modified Observer's Assessment of Alertness/Sedation (MOAA/S) Scale ≤ 3; T_MOAA/S = 5_, the time from stop for infusion of dexmedetomidine for D group or remimazolam for R group to reach MOAA/S = 5. ^*^: Phenyephrine (µg), 1.7 ± 7.1 in D group *vs.* 2.5 ± 8.5 in R group (*p* = 0.560). ^†^: Ephedrine (mg): 0.1 ± 0.7 in D group *vs.* 0.1 ± 0.8 in R group (*p* = 0.560).

**Table 2:** Postoperative pain and postoperative nausea and vomiting (PONV) at post-anesthetic care unit (PACU) stay.

		D group (n = 35)	R group (n = 35)	*P* value
Postoperative pain (0 - 100)				
	VAS at PACU0	0.9 ± 2.8	1.1 ± 3.2	0.692
	VAS at PACU5	0.9 ± 2.8	1.1 ± 3.2	0.692
	VAS at PACU10	0.9 ± 2.8	1.1 ± 3.2	0.692
	VAS at PACU15	1.4 ± 3.6	1.1 ± 3.2	0.731
	VAS at PACU20	1.4 ± 3.6	1.4 ± 3.6	0.456
	VAS at PACU25	1.4 ± 3.6	1.4 ± 3.6	0.456
	VAS at PACU30	1.7 ± 4.5	1.4 ± 3.6	0.784
Rescue for postoperative pain				
	Ketorolac (mg)	-	-	-
	Fentanyl (µg)	-	-	-
PONV incidence		3/35	3/35	1.000
PONV (0 - 3)				
	PONV at PACU0	0.0 ± 0.0 (0/35)	0.0 ± 0.0 (0/35)	1.000
	PONV at PACU5	0.0 ± 0.2 (2/35)	0.0 ± 0.2 (3/35)	0.645
	PONV at PACU10	0.0 ± 0.2 (2/35)	0.0 ± 0.2 (3/35)	0.645
	PONV at PACU15	0.1 ± 0.4 (3/35)	0.0 ± 0.2 (2/35)	0.626
	PONV at PACU20	0.1 ± 0.4 (2/35)	0.0 ± 0.2 (2/35)	0.626
	PONV at PACU25	0.1 ± 0.3 (1/35)	0.0 ± 0.1 (1/35)	0.984
	PONV at PACU30	0.0 ± 0.0 (0/35)	0.0 ± 0.0 (0/35)	1.000
Rescue for PONV				
	Ondansetron (mg)	-	-	-
	Dexamethasone (mg)	-	-	-

Data is expressed as mean ± standard deviation. **Abbreviations:** VAS, visual analogue pain scale; PACU0, on arrival at post-anesthetic care unit (PACU); PACU5, 5 minutes after arrival at PACU; PACU10, 10 minutes after arrival at PACU; PACU15, 15 minutes after arrival at PACU; PACU20, 20 minutes after arrival at PACU; PACU25, 25 minutes after arrival at PACU; PACU30, 30 minutes after arrival at PACU.

## References

[1] Bugada D, Ghisi D, Mariano ER (2017). Continuous regional anesthesia: a review of perioperative outcome benefits. Minerva Anestesiol.

[2] Fischer B (2010). Benefits, risks, and best practice in regional anesthesia: do we have the evidence we need?. Reg Anesth Pain Med.

[3] McKeage K, Perry CM (2003). Propofol: a review of its use in intensive care sedation of adults. CNS Drugs.

[4] De Cosmo G, Congedo E, Clemente A, Aceto P (2005). Sedation in PACU: the role of propofol. Curr Drug Targets.

[5] Silva-Jr JM, Katayama HT, Nogueira FAM, Moura TB, Alves TL, de Oliveira BW (2019). Comparison of dexmedetomidine and benzodiazepine for intraoperative sedation in elderly patients: a randomized clinical trial. Reg Anesth Pain Med.

[6] Sheta SA, Al-Sarheed MA, Abdelhalim AA (2014). Intranasal dexmedetomidine vs midazolam for premedication in children undergoing complete dental rehabilitation: a double-blinded randomized controlled trial. Paediatr Anaesth.

[7] Jo YY, Lee D, Jung WS, Cho NR, Kwak HJ (2016). Comparison of Intravenous Dexmedetomidine and Midazolam for Bispectral Index-Guided Sedation During Spinal Anesthesia. Med Sci Monit.

[8] Kim SH, Fechner J (2022). Remimazolam - current knowledge on a new intravenous benzodiazepine anesthetic agent. Korean J Anesthesiol.

[9] Lee HC (2022). Remimazolam: another option for induction of general anesthesia?. Korean J Anesthesiol.

[10] Jungquist CR, Quinlan-Colwell A, Vallerand A, Carlisle HL, Cooney M, Dempsey SJ (2020). American Society for Pain Management Nursing Guidelines on Monitoring for Opioid-Induced Advancing Sedation and Respiratory Depression: Revisions. Pain Manag Nurs.

[11] Pastis NJ, Hill NT, Yarmus LB, Schippers F, Imre M, Sohngen W (2022). Correlation of Vital Signs and Depth of Sedation by Modified Observer's Assessment of Alertness and Sedation (MOAA/S) Scale in Bronchoscopy. J Bronchology Interv Pulmonol.

[12] Faul F, Erdfelder E, Lang AG, Buchner A (2007). G*Power 3: a flexible statistical power analysis program for the social, behavioral, and biomedical sciences. Behav Res Methods.

[13] Faul F, Erdfelder E, Buchner A, Lang AG (2009). Statistical power analyses using G*Power 3.1: tests for correlation and regression analyses. Behav Res Methods.

[14] Lee A, Shirley M (2021). Remimazolam: A Review in Procedural Sedation. Drugs.

[15] Eren G, Cukurova Z, Demir G, Hergunsel O, Kozanhan B, Emir NS (2011). Comparison of dexmedetomidine and three different doses of midazolam in preoperative sedation. J Anaesthesiol Clin Pharmacol.

[16] Carollo DS, Nossaman BD, Ramadhyani U (2008). Dexmedetomidine: a review of clinical applications. Curr Opin Anaesthesiol.

[17] Arain SR, Ebert TJ (2002). The Efficacy, Side Effects, and Recovery Characteristics of Dexmedetomidine Versus Propofol When Used for Intraoperative Sedation. Anesthesia & Analgesia.

[18] Phan H, Nahata MC (2008). Clinical uses of dexmedetomidine in pediatric patients. Paediatr Drugs.

[19] Dai G, Pei L, Duan F, Liao M, Zhang Y, Zhu M (2021). Safety and efficacy of remimazolam compared with propofol in induction of general anesthesia. Minerva Anestesiol.

[20] Liu T, Lai T, Chen J, Lu Y, He F, Chen Y (2021). Effect of remimazolam induction on hemodynamics in patients undergoing valve replacement surgery: A randomized, double-blind, controlled trial. Pharmacol Res Perspect.

[21] Chen S, Wang J, Xu X, Huang Y, Xue S, Wu A (2020). The efficacy and safety of remimazolam tosylate versus propofol in patients undergoing colonoscopy: a multicentered, randomized, positive-controlled, phase III clinical trial. Am J Transl Res.

[23] Venn RM, Hell J, Grounds RM (2000). Respiratory effects of dexmedetomidine in the surgical patient requiring intensive care. Crit Care.

[24] Hu B, Jiang K, Shi W, Xiao S, Zhang S, Zhang Y (2022). Effect of Remimazolam Tosilate on Respiratory Depression in Elderly Patients Undergoing Gastroscopy: A Multicentered, Prospective, and Randomized Study. Drug Des Devel Ther.

[25] Kim KM (2022). Remimazolam: pharmacological characteristics and clinical applications in anesthesiology. Anesth Pain Med (Seoul).

